# Association between alendronate and atypical femur fractures: a meta-analysis

**DOI:** 10.1530/EC-14-0120

**Published:** 2015-02-04

**Authors:** Lu Liu, Chunyan Li, Peng Yang, Jian Zhu, Dongmei Gan, Le Bu, Manna Zhang, Chunjun Sheng, Hong Li, Shen Qu

**Affiliations:** Department of Endocrinology, Shanghai Tenth People's Hospital, Tongji University School of Medicine, 301 Yanchang Middle Road, Shanghai, 200072, China; 1 Department of Internal Medicine, Shanghai Dachang Hospital, Shanghai, 200442, China; 2 Department of Paediatrics, Ningbo Women and Children's Hospital, Ningbo, Zhejiang Province, 315012, China

**Keywords:** alendronate, atypical femur fractures, meta-analysis, osteoporosis

## Abstract

Alendronate (ALN) is a commonly used drug for the treatment of osteoporosis. Atypical femur fractures (AFFs) have been associated with long-term use of ALN and have recently become the subject of considerable attention as ALN use increases. This meta-analysis aimed to determine the relationship between ALN and AFF. The Embase, PubMed, and Cochrane library databases were searched for relevant studies published before November 6, 2014. Studies clearly reporting the relationship between ALN and AFF were selected for our analysis. From these results, the relationship between ALN and AFF was analyzed. Weighted mean differences were calculated using a random-effects model. Five studies were included in this meta-analysis. The results revealed that the use of ALN will not increase the risk of AFF in short term (*P*>0.05), but there will be a risk of AFF (*P*<0.05) with long-term (>5 years) use of ALN. These findings indicate that long-term use of ALN is a risk factor for AFF and that more attention should be paid to the clinical applications of ALN.

## Introduction

Osteoporosis is a major metabolic bone disease that affects 44 million Americans or 55% of the population at the age of 50 years or older. Out of them, ten million individuals already have the disease while 34 million more are at an increasingly high risk of osteoporosis (1). Though osteoporosis is often thought of as an older person's disease, it can strike at any age. The disease causes a significant amount of morbidity and mortality in patients and is often diagnosed after a fracture occurs. Current medications used to treat osteoporosis include bisphosphonates, raloxifene, calcitonin, and hormone replacement therapy (2). Bisphosphonates are a class of widely prescribed drugs that are proven to be effective in reducing common bone fractures in people with osteoporosis and those at a high risk of fractures (3, 4, 5).

Alendronate (ALN) is a potent oral bisphosphonate with a prolonged duration of action and is the most commonly prescribed bisphosphonate (6). The pharmacokinetics of ALN allow for a once-weekly regimen that leads to continued maintenance of bone mineral density for months to years after discontinuation (7). ALN could inhibit osteoclast-mediated bone resorption and normalize the rate of bone turnover to premenopausal levels. In both animal and human studies, administration of ALN could increase bone mass and maintain histologically normal bone (8, 9, 10). Bone *et al*. (11) found that clinical symptoms and indicators improved significantly with long-term use of ALN for elderly postmenopausal women with osteoporosis. However, results from a growing number of recent studies have indicated that bone turnover will be suppressed excessively with long-term use of ALN, which will lead to the occurrence of atypical femur fracture (AFF) (12). This study collected relevant literature on the use of ALN and the consequent incidence of AFF and utilized meta-analysis to clarify the relationship between ALN use and AFF occurrence to provide credible advice for clinicians.

## Methods

### Literature retrieval

The Cochrane library, PubMed, and Embase databases were searched to retrieve relevant studies published before November 6, 2014. The search criteria ‘femoral fracture’ or ‘femur fracture’ or ‘hip fracture’ or ‘diaphyseal’ or ‘atypical fractures’ and ‘ALN’ were used in text word searches, while the ‘related articles’ function was used to broaden the search. Reference lists of selected articles were also manually examined to find relevant studies not discovered during the database searches. Any observational or interventional studies that examined the relationship between ALN and AFF were selected. All titles, abstracts, and full papers of potentially relevant studies were assessed for eligibility based on predefined inclusion and exclusion criteria. Eligible papers included i) studies on the use of ALN and AFF that were published before November 6, 2014, ii) cohort studies to confirm osteoporosis in a population, iii) studies where the statistical indicator was AFF. Finally, relevant articles were examined to ensure that the diagnosis standards of AFF were consistent. When several reports from the same study were published, only the most recent or informative was included in our meta-analysis. The language was restricted to English.

### Data extraction

Data extractions of all variables and outcomes of interest and assessment of methodological quality were performed independently by two readers. Any disagreements were resolved through discussion to reach a consensus. The methodological quality of the trials was evaluated using the assessment forms from the Agency for Healthcare Research and Quality.

### Statistical analysis

Statistical analysis was performed using the ReviewManager 5.0 Software (Cochrane Collaboration, Nordic Cochrane Centre, Copenhagen, Denmark). Continuous variables were analyzed using the weighted mean difference. *P* values <0.05 were considered statically significant, and 95% CIs were reported. Homogeneity was tested using the *Q* statistic (significance level at *P*<0.10) and the *I* ^2^ statistic (significance level at *I* ^2^>50%). If the overall effects of multiple findings were consistent, the fixed-effects model was used; otherwise, the random-effects model was employed. The presence of publication bias was assessed by the visual inspection of a funnel plot.

## Results

### Literature search

**Figure 1:**
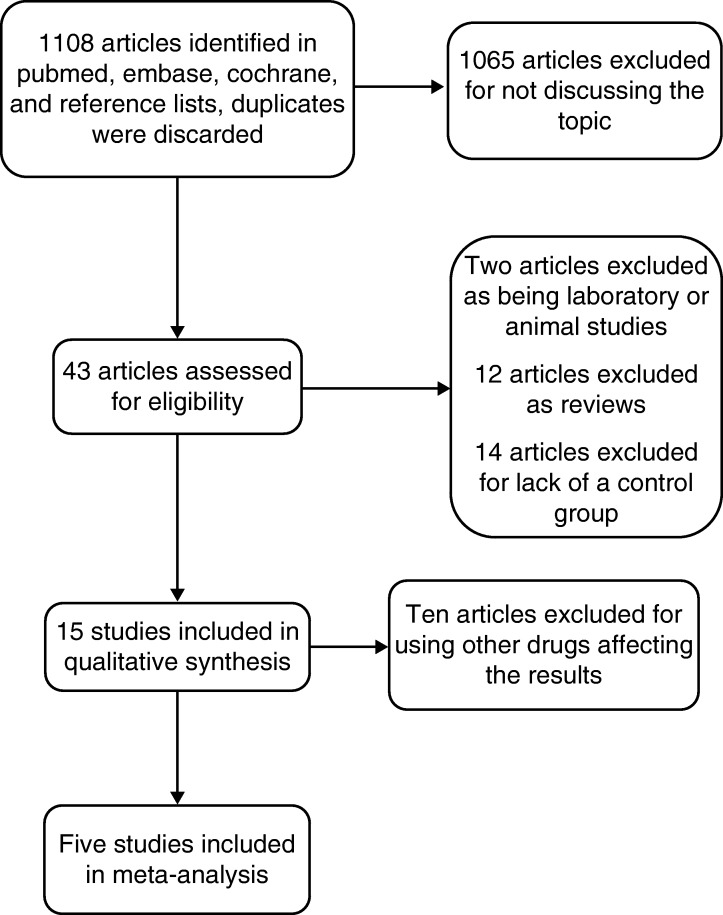
Search strategy flow diagram.

The initial literature search retrieved 1108 relevant articles (duplicates were discarded). Of these articles, 1065 were excluded from our analysis for not investigating the topic of interest. The abstracts were reviewed from the 43 remaining articles, and another 38 articles were excluded (two laboratory or animal studies, 12 reviews, 14 without a control group, and ten with other bisphosphonates). Therefore, five studies matched the selection criteria and were suitable for our meta-analysis (13, 14, 15, 16, 17). A flow-diagram for the selection of studies included in our meta-analysis is shown in Fig. 1. A total of 231 203 patients (53 134 experimental group and 178 069 control group) were included in our analysis. The key characteristics of the included studies are summarized in Table 1. Table 2 summarizes the methodological quality of the studies.

### Main analysis

**Figure 2:**
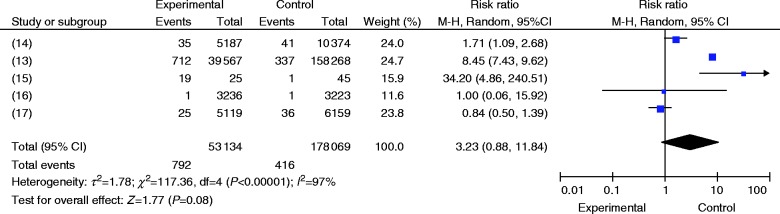
The risk of AFF for patients using ALN.

**Figure 3:**
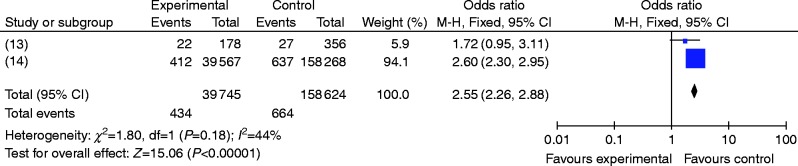
The risk of AFF for patients using ALN for a long period (≥5 years).

Figure 2 summarizes the outcome of our meta-analysis. Heterogeneity analysis was performed on the five papers, and the results indicated that heterogeneity was significant (*P*<0.05, *I* ^2^=97%). Therefore, the random-effects model was employed. The results were RR=3.23, 95% CI (0.88, 11.84), *P*>0.05 (Fig. 2), and the differences were not statistically significant. Among them, 5-year oral administration periods were considered in two studies (13, 14), and further stratified studies were conducted. Heterogeneity analysis was also performed on these two papers, and the results indicated that heterogeneity was not significant; therefore, the fixed-effects model was used (*P*=0.18, *I* ^2^=44%). The results were RR=2.55, 95% CI (2.26, 2.88), *P*<0.05 (Fig. 3), and the difference was statistically significant (as shown in Fig. 3).

## Discussion

ALN belongs to the third generation of bisphosphonate drugs that could inhibit the activity of osteoclasts by physicochemically combining with the bone matrix and subsequently blocking the action of osteoclasts by inducing the secretion of a variety of cytokines. In addition, ALN can regulate the metabolism of calcium *in vivo*, prevent the loss of bone mass, and augment bone mineral density, all of which explain why ALN is the most widely used bisphosphonate. Bisphosphonates are the primary agents used to treat osteoporosis, metastatic bone malignancies, Paget's disease, multiple myeloma, and hypercalcemia in malignancy. Moreover, bisphosphonates are commonly used for prevention and treatment of a variety of other skeletal conditions, such as low bone density and osteogenesis imperfecta (18, 19).

AFF, the rare adverse reaction resulting from long-term use of ALN, has recently become the subject of more attention from clinicians. Odvina *et al*. (20) were the first, to our knowledge, to report AFF from ALN use. They found that spontaneous nonvertebral fractures can occur in patients undergoing long-term use of ALN, even in the absence of obvious initiating trauma. Furthermore, these patients exhibited postoperative delayed union or nonunion. Since then, similar clinical cases have been reported at different medical centers (21, 22, 23). According to the existing data, the incidence rate of AFF is <4‰ in high femoral fractures, and most of the literatures related to AFF were case reports (as shown in Table 3).

Large, randomized, controlled trials have demonstrated that ALN therapy for 3–4 years is effective in reducing the risk of both nonvertebral and vertebral fractures in osteoporotic women (16). However, there is considerable controversy over the ideal duration of antiresorptive therapy in the light of reports regarding ALN-related AFF. Two randomized trials have been implemented to assess the efficacy of long-term use of ALN and the risk of fractures (24, 25). The results indicated that women are at a very high risk of clinical vertebral fractures when ALN was administered for more than 5 years. However, these trials were conducted in post-menopausal women, and therefore the results may not apply to younger women or to men.

To clarify the relationship between ALN and AFF, and provide credible advice for clinicians, we performed this meta-analysis. The results indicated that ALN administration did not increase the risk of AFF in short term (lower than 5 years), but there will be a risk of AFF (*P*<0.05) with long-term (>5 years) use of ALN.

The mechanisms of AFF are still unclear. Allen *et al*. (26) found that the bone micro-damage caused by ALN increased by more than sevenfold compared with the controls, with a concurrent bone mineral density decrease of 40%, leading to increased ease of fracture. Results from another study also carried out by Allen (27) indicated that the long-term use of ALN could exert adverse effects on bone trabeculae and heterogeneous cross-linking of collagen, which will lead to bone fragility. The bone turnover and metabolism of patients with osteoporosis were abnormal, which represents a risk factor *per se* for the occurrence of AFF (28). Results from some studies have indicated that bone turnover could be suppressed excessively with long-term use of ALN, which will lead to excessive accumulation of bone micro-damage and the occurrence of AFF (21). In short, further animal experiments and clinical studies should be conducted to clarify the mechanism of AFF.

Considering the potential for the increased risk of AFF, we think that the use of ALN should not exceed 5 years, and it will be appropriate for patients to discontinue the use of ALN after 5 years. Moreover, most of the experts also recommend that the application of ALN should not exceed 5 years. AFF could heal themselves after discontinuation of ALN treatment, but can quickly recur when treatment resumes (23). Teriparatide (recombinant parathyroid peptide) is recommended for patients with AFF. In theory, teriparatide could promote favorable bone metabolism in patients with AFF; however, it is still unclear whether bone mineral density remains unchanged during the period of teriparatide treatment, or how long the antiresorptive drugs should be taken (29).

However, our research has some limitations. Of the five studies included, two were conducted in Denmark, one in Taiwan, while the others were conducted in America, leading to clinical heterogeneity in our study. Moreover, there is little data available regarding the long-term use of ALN and AFF, which may affect our results. In the future, multi-center prospective cohort studies using large sample sizes and various subgroups according to sex and age are needed.

The American Society for Bone and Mineral Research proposed that physicians should assess the condition of each patient individually because the optimal length of bisphosphonate therapy remains unknown and must be considered on a case-by-case basis (30). Objectively speaking, AFF is the only rare adverse reaction caused by ALN. As such, ALN is still a better option for patients with osteoporosis, and the benefits outweigh the possible risks for the majority of patients. Based on this approach, clinicians should not reject ALN outright, but should pay attention to its application, particularly the duration of administration.

## Conclusion

In summary, this meta-analysis suggests that patients undergoing long-term treatment using ALN may be at an increased risk of AFF.

## Figures and Tables

**Table 1:** Characteristics of the studies included in this research.

**Reference**	**Research on life**	**Study area**	**Study design**	**Female patients** (%)	**Sample size**	**Age**	**AFF number**	**AFF type**
(13)	1997–2005	Denmark	Cohort study	91.2	10 374 vs 5187	73.1±8.5 vs 73.1±8.5	76	Trochanter, femoral shaft
(14)	1996–2005	Denmark	Cohort study	82.8	158 268 vs 39 567	69.8±11.6 vs 69.8±11.6	1049	Trochanter, femoral shaft
(15)	2002–2007	America	Cohort study	84.3	45 vs 25	77.1 vs 69.4	20	Trochanter, femoral shaft
(16)	Unknown	America	Cohort study	100	3223 vs 3236	Unknown	2	Trochanter, femoral shaft
(17)	2001–2005	Taiwan	Cohort study	Unknown	6159 vs 5119	Unknown	61	Trochanter, femoral shaft

**Table 2:** The methodological quality of the studies included in this research.

**Criterion**	**(13)**	**(14)**	**(15)**	**(16)**	**(17)**
1. Defined the source of information (survey and record review)	Yes	Yes	Yes	Yes	Yes
2. Listed inclusion and exclusion criteria for exposed and unexposed subjects (cases and controls) or referred to previous publications	Yes	Yes	Yes	Yes	Yes
3. Indicated time period used for identifying patients	Yes	Yes	Yes	No	Yes
4. Indicated whether or not subjects were consecutive if not populationed-based	No	No	No	Yes	Yes
5. Indicated if evaluators of subjective components of study were masked to other aspects of the participants	Unclear	Unclear	Unclear	Unclear	Unclear
6. Described any assessments undertaken for quality assurance purposes	Yes	Yes	Yes	Yes	Yes
7. Explained any patient exclusions from analysis	Yes	Yes	Yes	Yes	No
8. Described how confounding was assessed and/or controlled	Yes	Yes	Yes	Yes	Yes
9. If applicable, explained how missing data were handled in the analysis	Yes	Yes	Yes	Yes	Yes
10. Summarised patient response rates and completeness of data collection	Yes	Yes	Yes	Yes	Yes
11. Clarified what follow-up, if any, was expected and the percentage of patients for which incomplete data or follow-up was obtained	Yes	Yes	Yes	Yes	Yes

**Table 3:** Case studies of atypical femur fractures related to bisphosphonates.

**Reference**	**AFF number**	**Average treatment time**	**Average age**
(20)	7	5.6 (3–8)	60.0 (49–68)
(28)	9	4.2 (2.5–5)	66.9 (55–82)
(31)	26	4.4 (2–10)	66.1 (53–82)
(32)	12	7.3 (5.5–9)	70.4 (55–83)
(15)	19	6.9	69.5
(33)	2	7.0	72.0
(34)	2	9.0	57.0
(2)	14	8.6 (5–13)	61.0 (53–75)
(35)	3	9.0	66.5
(36)	3	7.3 (6–9)	63.3 (59–66)
(12)	2	6.0	60.0
(37)	16	4.5 (2–7)	68.0 (53–92)
(38)	59	7.1 (4–11)	73.7 (67–85)
